# 6-Hy­droxy-2,5,7,8-tetra­methyl-3,4-dihydro-2*H*-1-benzopyran-2-carbonitrile, from synchrotron data

**DOI:** 10.1107/S1600536811025517

**Published:** 2011-07-06

**Authors:** Krzysztof Brzezinski, Aneta Baj, Piotr Wałejko, Stanisław Witkowski, Zbigniew Dauter

**Affiliations:** a Synchrotron Radiation Research Section, MCL, National Cancer Institute, Argonne National Laboratory, Biosciences Division, Bldg. 202, Argonne, IL 60439, USA; b Institute of Chemistry, University of Białystok, Piłsudskiego 11/4, 15-443 Białystok, Poland

## Abstract

The crystal structure of the title compound, C_14_H_17_NO_2_, solved and refined against synchrotron diffraction data, contains one formula unit in an asymmetric unit. In the crystal, mol­ecules form right-handed helices located at the 2_1_ screw axis parallel to the *a*-axis direction, generated by O—H⋯N hydrogen bonding between the hy­droxy group and carbonitrile group of an adjacent mol­ecule.

## Related literature

For background to the chemistry of chroman compounds and their applications as anti­oxidants and anti-inflammatory agents, see Cohen *et al.* (1989 ▶); van Acker *et al.* (1993 ▶); Boscoboinik *et al.* (1995 ▶). For the preparation of nitriles from primary amides, see: Campagna *et al.* (1977 ▶).
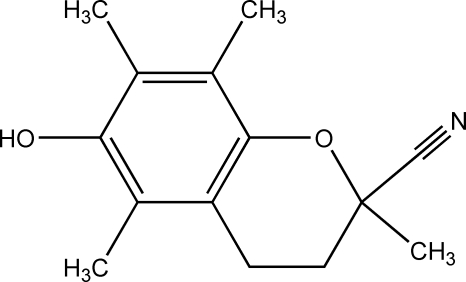


## Experimental

### 

#### Crystal data

C_14_H_17_NO_2_
*M* *_r_* = 231.29
Orthorhombic, 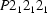

*a* = 5.890 (5) Å
*b* = 10.30 (1) Å
*c* = 19.710 (19) Å
*V* = 1195.7 (19) Å^3^
*Z* = 4
Synchrotron radiation
λ = 0.75000 Å
μ = 0.09 mm^−1^
*T* = 100 K
0.1 × 0.03 × 0.01 mm

#### Data collection

MAR 300 CCD diffractometer
Absorption correction: multi-scan (*SCALEPACK*; Otwinowski *et al.*, 2003 ▶) *T*_min_ = 0.991, *T*_max_ = 0.999
15411 measured reflections
1818 independent reflections
1815 reflections with *I* > 2σ(*I*)
*R*_int_ = 0.031

#### Refinement

*R*[*F* ^2^ > 2σ(*F* ^2^)] = 0.032
*wR*(*F* ^2^) = 0.089
*S* = 1.00
1818 reflections
158 parameters
H-atom parameters constrained
Δρ_max_ = 0.27 e Å^−3^
Δρ_min_ = −0.20 e Å^−3^

### 

Data collection: *SER-CAT APS beamline software*; cell refinement: *HKL-2000* (Otwinowski & Minor, 1997 ▶); data reduction: *HKL-2000*; program(s) used to solve structure: *SHELXD* (Sheldrick, 2008 ▶); program(s) used to refine structure: *SHELXL97* (Sheldrick, 2008 ▶); molecular graphics: *ORTEP-3* (Farrugia, 1997 ▶) and *pyMOL* (DeLano, 2002 ▶); software used to prepare material for publication: *SHELXL97*.

## Supplementary Material

Crystal structure: contains datablock(s) global, I. DOI: 10.1107/S1600536811025517/kp2339sup1.cif

Structure factors: contains datablock(s) I. DOI: 10.1107/S1600536811025517/kp2339Isup2.hkl

Supplementary material file. DOI: 10.1107/S1600536811025517/kp2339Isup3.cml

Additional supplementary materials:  crystallographic information; 3D view; checkCIF report

## Figures and Tables

**Table 1:** Hydrogen-bond geometry (Å, °)

*D*—H⋯*A*	*D*—H	H⋯*A*	*D*⋯*A*	*D*—H⋯*A*
O15—H15⋯N13^i^	0.84	2.23	3.013 (2)	155

Symmetry code: (i) 
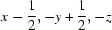
.

**Fig. 1.**
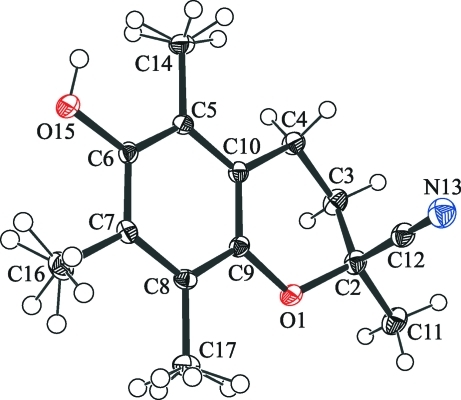
The molecular structure of the title compound. Displacement ellipsoids are drawn at the 50% probability level.

**Fig. 2.**
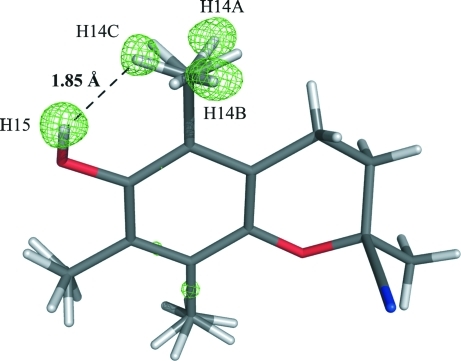
The OMIT map at hydrogen atoms bounded to O15 and C14 (conformation occupied by H14A—C atoms) contoured at 0.21 e Å-3 (4.5 σ level, green). Dashed line represents a short intramolecular hydrogen–hydrogen contact.

**Fig. 3.**
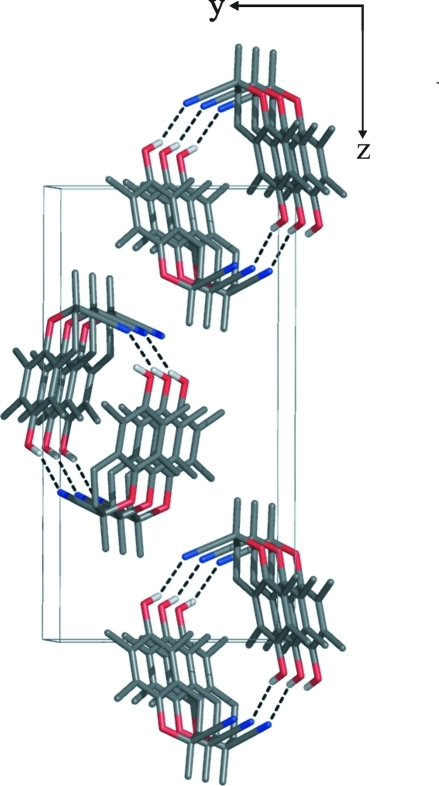
The packing diagram viewed along a axis. Dashed lines represent hydrogen bonds. For clarity, only hydrogen atoms engaged in hydrogen bond formation are shown.

**Fig. 4.**
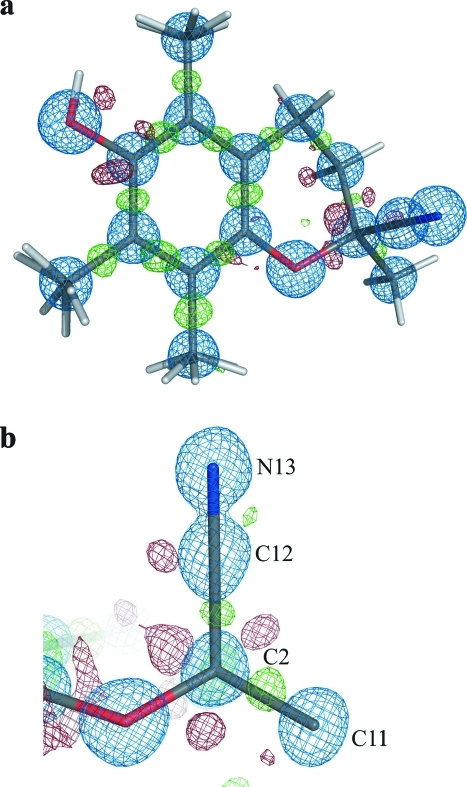
The deformation of electron distribution revealed in difference Fourier synthesis; (a) The Fobs and difference syntheses for a title compound contoured at 2.27 e Å3 (2.0 σ level, blue) and ±0.13 e Å-3 (±3.5 σ level, green and red), respectively; (b) The Fobs and difference syntheses contoured at 2.27 e Å-3 (2.0 σ level, blue), ±0.11 e Å3 (±3.0 σ level, green and red), respectively, hydrogen atoms are omitted.

## supplementary crystallographic information

### Comment

2-Substituded chromans (3,4-dihydro-2*H*-1-benzopyrans) are an important class of compounds possessing significant biological properties (Cohen *et al.*, 1989). The title compound is potentially more active as an antioxidant than 6-hydroxy-2,2,5,7,8-pentamethylchroman, the model compound of α-tocopherol (Van Acker *et al.*, 1993). Carbonitrile derivatives appear to inhibit smooth muscle cell proliferation by the non-antioxidant mechanism (Boscoboinik *et al.*, 1995).

The asymmetric unit contains one molecule of the title compound (Fig. 1). The crystal, formed spontaneously, contains a homochiral molecule (not established experimentally). Hydrogen atoms of C14, C16 and C17 methyl groups are partially disorded and are modelled in two alternative conformations. For one conformation of the C14 methyl group, notable is a short intramolecular interaction between hydroxy H15 and H14C atoms. Such a short distance could indicate some refinement problem but on the other hand problematic hydrogen atom positions are clearly confirmed by *OMIT* map (Fig. 2). The hydrogen bond network is based on the interaction between hydroxy group and carbonitrile group of a subsequent molecule. Such interactions generate the right handed helices located at the 2_1_ screw axis parallel to *a* direction (Fig. 3). A high quality diffraction data revealed a detailed electron density distribution among a molecule of the title compound. The difference Fourier synthesis indicates clearly a presence of valence electrons for most of covalent bonds (Fig. 4a). This is especially noticeable for tetrahedral deformation of electron density distribution at C2 atom and its neighbourhood (Fig. 4 b). In this case positive peaks are located on covalent bonds and correspond to valence electrons. Negative peaks are positioned in a regular and symmetrical manner indicating the electron density shift in the bond direction.

### Experimental

Anhydrous pyridine (26 µ*L*, 0.32 mmol) and trifluoroacetic anhydride (25 µ*L*, 0.18 mmol) were added dropwise to *rac*-6-hydroxy- 2,5,7,8-tetramethylchroman-2-carboxamide (40 mg, 0.16 mmol) solution in dry THF (2 mL) at 273 K. The reaction mixture was warmed to room temperature and further stirred for 18 h. The reaction mixture was diluted with CHCl_3_, washed with water and brine. The organic layer was dried over anhydrous Na_2_SO_4_, filtered off and concentrated *in vacuo*. The crude product was purified by flash column chromatography (hexane/ethyl acetate 85:15) to give the title compound as a tan solid (15.2 mg, 41% yield). *M*.p. 423–425 K (from ethyl acetate/hexane *v*/*v* 1:2); ^1^H NMR (400 MHz, CDCl_3_): δ 4.34 (s, 1H), 2.93 (ddd, *J* = 17.6, 12.3 and 6.6 Hz, 1H), 2.77 (ddd, *J* = 17.0, 6.2 and 1.7 Hz, 1H), 2.31 (ddd, *J* = 13.8, 6.6 and 2.0 Hz, 1H), 2.17 (s, 3H), 2.13 (s, 6H), 1.96 (ddd, *J* = 13.8, 12.3 and 6.2 Hz, 1H), 1.83 (s, 6H); ^13^C NMR (100 MHz, CDCl_3_): δ 146.3, 144.1, 122.9, 121.7, 120.0, 118.6, 116.5, 69.8, 32.6, 26.9, 21.2, 12.1, 11.8, 11.3; IR (KBr) ν_max_/cm^-1^: 3527, 2930, 2235 (–CN), 1463, 1261; ESI-MS, m/*z*: 254 [MNa^+^, 100%]. The crystallization was carried out at room temperature by slow evaporation of the racemic mixture of 3,4-dihydro-6-hydroxy-2,5,7,8-tetramethyl-2*H*-1-benzopyran-2-carbonitrile solution in acetone yielding homochiral crystals (chiral resolution).

### Refinement

The absolute configuration at C2 has not been established. Hydrogen atoms were constrained to idealised positions with C—H distances fixed at 0.98–0.99 Å and *U*_iso_(H) = 1.5*U*_eq_(C) for methyl and hydroxy hydrogen atoms and 1.2*U*_eq_(C) for methylene ones. The sum of occupancies of alternative positions of disordered atoms of was constrained to unity.

### Crystal data

**Table:**

C_14_H_17_NO_2_	*F*(000) = 496
*M**_r_* = 231.29	*D*_x_ = 1.285 Mg m^−^^3^
Orthorhombic, *P*2_1_2_1_2_1_	Synchrotron radiation, λ = 0.75000 Å
Hall symbol: P 2ac 2ab	Cell parameters from 1841 reflections
*a* = 5.890 (5) Å	θ = 2.2–30.9°
*b* = 10.30 (1) Å	µ = 0.09 mm^−^^1^
*c* = 19.710 (19) Å	*T* = 100 K
*V* = 1195.7 (19) Å^3^	Needle, colourless
*Z* = 4	0.1 × 0.03 × 0.01 mm

### Data collection

**Table:**

MAR 300 CCD diffractometer	1818 independent reflections
Radiation source: SER-CAT 22-ID synchrotron beamline APS, USA	1815 reflections with *I* > 2σ(*I*)
Si111 double crystal	*R*_int_ = 0.031
ω scans	θ_max_ = 30.9°, θ_min_ = 2.2°
Absorption correction: multi-scan (*SCALEPACK*; Otwinowski *et al.*, 2003)	*h* = 0→8
*T*_min_ = 0.991, *T*_max_ = 0.999	*k* = 0→14
15411 measured reflections	*l* = 0→26

### Refinement

**Table:**

Refinement on *F*^2^	Primary atom site location: structure-invariant direct methods
Least-squares matrix: full	Secondary atom site location: difference Fourier map
*R*[*F*^2^ > 2σ(*F*^2^)] = 0.032	Hydrogen site location: inferred from neighbouring sites
*wR*(*F*^2^) = 0.089	H-atom parameters constrained
*S* = 1.00	*w* = 1/[σ^2^(*F*_o_^2^) + (0.0505*P*)^2^ + 0.297*P*] where *P* = (*F*_o_^2^ + 2*F*_c_^2^)/3
1818 reflections	(Δ/σ)_max_ < 0.001
158 parameters	Δρ_max_ = 0.27 e Å^−^^3^
0 restraints	Δρ_min_ = −0.20 e Å^−^^3^

### Special details

**Table:**

Experimental. The crystal was mounted with vaseline on a pin-attached capillary. Upon mounting, the crystal was quenched to 100 K in a nitrogen-gas stream supplied by an Oxford Cryo-Jet. Diffraction data were measured at the station 22-ID of the APS synchrotron by rotation method.
Geometry. All e.s.d.'s are estimated using the full covariance matrix. The cell e.s.d.'s are taken into account individually in the estimation of e.s.d.'s in distances, angles and torsion angles; correlations between e.s.d.'s in cell parameters are only used when they are defined by crystal symmetry. An approximate (isotropic) treatment of cell e.s.d.'s is used for estimating e.s.d.'s involving l.s. planes.
Refinement. Refinement of *F*^2^ against all reflections. The weighted *R*-factor *wR* and goodness of fit *S* are based on *F*^2^, conventional *R*-factors *R* are based on *F*, with *F* set to zero for negative *F*^2^. The threshold expression of *F*^2^ > 2σ(*F*^2^) is used only for calculating *R*-factors *etc*. and is not relevant to the choice of reflections for refinement. *R*-factors based on *F*^2^ are statistically about twice as large as those based on *F*.

### Fractional atomic coordinates and isotropic or equivalent isotropic displacement parameters (Å^2^)

**Table:**

	*x*	*y*	*z*	*U*_iso_*/*U*_eq_	Occ. (<1)
O1	0.64100 (17)	0.44132 (9)	0.20022 (4)	0.0181 (2)	
C2	0.5481 (2)	0.32600 (13)	0.22950 (6)	0.0185 (3)	
C3	0.2980 (2)	0.31287 (15)	0.21136 (6)	0.0216 (3)	
H3A	0.2131	0.3877	0.2301	0.026*	
H3B	0.2363	0.2328	0.2322	0.026*	
C4	0.2641 (2)	0.30738 (13)	0.13455 (6)	0.0189 (2)	
H4A	0.2914	0.2176	0.1186	0.023*	
H4B	0.1049	0.3302	0.1237	0.023*	
C5	0.3920 (2)	0.42209 (11)	0.02739 (6)	0.0139 (2)	
C6	0.5435 (2)	0.50415 (11)	−0.00586 (6)	0.0139 (2)	
C7	0.7196 (2)	0.56738 (11)	0.02858 (6)	0.0146 (2)	
C8	0.7490 (2)	0.54480 (11)	0.09804 (6)	0.0143 (2)	
C9	0.6003 (2)	0.45959 (12)	0.13083 (5)	0.0141 (2)	
C10	0.4216 (2)	0.39890 (11)	0.09736 (6)	0.0141 (2)	
C11	0.5889 (3)	0.33520 (14)	0.30557 (6)	0.0246 (3)	
H11A	0.5030	0.4086	0.3241	0.037*	
H11B	0.7511	0.3482	0.3142	0.037*	
H11C	0.5387	0.2547	0.3274	0.037*	
C12	0.6795 (2)	0.21322 (13)	0.20181 (6)	0.0190 (3)	
N13	0.7821 (2)	0.12765 (12)	0.18050 (6)	0.0254 (3)	
C14	0.2015 (2)	0.35690 (13)	−0.01042 (6)	0.0205 (3)	
H14A	0.0587	0.3702	0.0141	0.031*	0.66 (2)
H14B	0.2327	0.2637	−0.0140	0.031*	0.66 (2)
H14C	0.1891	0.3944	−0.0560	0.031*	0.66 (2)
H14D	0.2646	0.2943	−0.0428	0.031*	0.34 (2)
H14E	0.1131	0.4224	−0.0349	0.031*	0.34 (2)
H14F	0.1028	0.3115	0.0218	0.031*	0.34 (2)
O15	0.52747 (19)	0.52971 (9)	−0.07450 (4)	0.0209 (2)	
H15	0.4404	0.4753	−0.0927	0.031*	
C16	0.8761 (2)	0.65795 (13)	−0.00891 (7)	0.0212 (3)	
H16A	0.8337	0.6602	−0.0570	0.032*	0.68 (2)
H16B	1.0328	0.6272	−0.0045	0.032*	0.68 (2)
H16C	0.8635	0.7454	0.0103	0.032*	0.68 (2)
H16D	0.7888	0.7077	−0.0423	0.032*	0.32 (2)
H16E	0.9937	0.6075	−0.0322	0.032*	0.32 (2)
H16F	0.9475	0.7177	0.0234	0.032*	0.32 (2)
C15	0.9388 (2)	0.61130 (12)	0.13588 (6)	0.0189 (2)	
H17A	0.9342	0.5857	0.1837	0.028*	0.85 (3)
H17B	0.9205	0.7056	0.1323	0.028*	0.85 (3)
H17C	1.0849	0.5858	0.1162	0.028*	0.85 (3)
H17D	0.9953	0.5538	0.1717	0.028*	0.15 (3)
H17E	0.8819	0.6918	0.1562	0.028*	0.15 (3)
H17F	1.0624	0.6315	0.1043	0.028*	0.15 (3)

### Atomic displacement parameters (Å^2^)

**Table:**

	*U*^11^	*U*^22^	*U*^33^	*U*^12^	*U*^13^	*U*^23^
O1	0.0230 (4)	0.0199 (4)	0.0114 (4)	−0.0028 (4)	−0.0020 (4)	0.0008 (3)
C2	0.0221 (6)	0.0208 (6)	0.0125 (5)	0.0001 (5)	0.0020 (4)	0.0019 (4)
C3	0.0197 (6)	0.0291 (6)	0.0161 (5)	−0.0006 (6)	0.0032 (5)	0.0042 (5)
C4	0.0177 (5)	0.0226 (6)	0.0164 (5)	−0.0046 (5)	0.0003 (5)	0.0033 (4)
C5	0.0156 (5)	0.0128 (5)	0.0134 (5)	0.0006 (4)	−0.0004 (4)	−0.0012 (4)
C6	0.0186 (5)	0.0121 (5)	0.0112 (5)	0.0014 (4)	0.0005 (4)	0.0001 (4)
C7	0.0166 (6)	0.0119 (5)	0.0154 (5)	0.0001 (4)	0.0022 (4)	0.0003 (4)
C8	0.0142 (5)	0.0135 (5)	0.0154 (5)	0.0004 (4)	−0.0001 (4)	−0.0031 (4)
C9	0.0165 (5)	0.0148 (5)	0.0109 (4)	0.0018 (5)	−0.0001 (4)	−0.0006 (4)
C10	0.0145 (5)	0.0144 (5)	0.0133 (5)	0.0002 (4)	0.0011 (4)	0.0006 (4)
C11	0.0305 (7)	0.0306 (7)	0.0128 (5)	0.0027 (6)	0.0004 (5)	0.0017 (5)
C12	0.0217 (6)	0.0217 (6)	0.0136 (5)	−0.0018 (5)	0.0003 (5)	0.0024 (4)
N13	0.0292 (6)	0.0258 (6)	0.0212 (5)	0.0014 (5)	0.0015 (5)	0.0008 (4)
C14	0.0210 (6)	0.0218 (6)	0.0187 (5)	−0.0054 (5)	−0.0039 (5)	−0.0012 (5)
O15	0.0296 (5)	0.0211 (4)	0.0119 (4)	−0.0058 (4)	−0.0021 (4)	0.0021 (3)
C16	0.0224 (6)	0.0187 (5)	0.0224 (5)	−0.0052 (5)	0.0034 (5)	0.0030 (5)
C15	0.0172 (5)	0.0191 (5)	0.0205 (5)	−0.0019 (5)	−0.0023 (5)	−0.0037 (4)

### Geometric parameters (Å, °)

**Table:**

O1—C9	1.4011 (18)	C11—H11B	0.9800
O1—C2	1.4296 (18)	C11—H11C	0.9800
C2—C12	1.499 (2)	C12—N13	1.148 (2)
C2—C11	1.521 (2)	C14—H14A	0.9800
C2—C3	1.522 (2)	C14—H14B	0.9800
C3—C4	1.528 (2)	C14—H14C	0.9800
C3—H3A	0.9900	C14—H14D	0.9800
C3—H3B	0.9900	C14—H14E	0.9800
C4—C10	1.5121 (18)	C14—H14F	0.9800
C4—H4A	0.9900	O15—H15	0.8400
C4—H4B	0.9900	C16—H16A	0.9800
C5—C6	1.3928 (18)	C16—H16B	0.9800
C5—C10	1.4103 (19)	C16—H16C	0.9800
C5—C14	1.5050 (19)	C16—H16D	0.9800
C6—O15	1.3815 (18)	C16—H16E	0.9800
C6—C7	1.4006 (18)	C16—H16F	0.9800
C7—C8	1.400 (2)	C15—H17A	0.9800
C7—C16	1.5051 (19)	C15—H17B	0.9800
C8—C9	1.3981 (18)	C15—H17C	0.9800
C8—C15	1.5080 (19)	C15—H17D	0.9800
C9—C10	1.3909 (18)	C15—H17E	0.9800
C11—H11A	0.9800	C15—H17F	0.9800
			
C9—O1—C2	116.12 (10)	C5—C14—H14E	109.4
O1—C2—C12	107.40 (12)	H14A—C14—H14E	72.0
O1—C2—C11	106.61 (11)	H14B—C14—H14E	137.6
C12—C2—C11	109.00 (11)	H14C—C14—H14E	40.3
O1—C2—C3	110.46 (11)	H14D—C14—H14E	109.5
C12—C2—C3	110.22 (12)	C5—C14—H14F	109.5
C11—C2—C3	112.95 (11)	H14A—C14—H14F	40.3
C2—C3—C4	111.25 (11)	H14B—C14—H14F	72.0
C2—C3—H3A	109.4	H14C—C14—H14F	137.6
C4—C3—H3A	109.4	H14D—C14—H14F	109.5
C2—C3—H3B	109.4	H14E—C14—H14F	109.5
C4—C3—H3B	109.4	C6—O15—H15	109.5
H3A—C3—H3B	108.0	C7—C16—H16A	109.5
C10—C4—C3	112.16 (11)	C7—C16—H16B	109.5
C10—C4—H4A	109.2	H16A—C16—H16B	109.5
C3—C4—H4A	109.2	C7—C16—H16C	109.5
C10—C4—H4B	109.2	H16A—C16—H16C	109.5
C3—C4—H4B	109.2	H16B—C16—H16C	109.5
H4A—C4—H4B	107.9	C7—C16—H16D	109.4
C6—C5—C10	118.93 (11)	H16A—C16—H16D	37.3
C6—C5—C14	121.01 (12)	H16B—C16—H16D	136.3
C10—C5—C14	120.05 (11)	H16C—C16—H16D	75.0
O15—C6—C5	122.20 (11)	C7—C16—H16E	109.5
O15—C6—C7	115.88 (11)	H16A—C16—H16E	74.9
C5—C6—C7	121.92 (12)	H16B—C16—H16E	37.3
C8—C7—C6	119.24 (11)	H16C—C16—H16E	136.2
C8—C7—C16	120.50 (12)	H16D—C16—H16E	109.5
C6—C7—C16	120.26 (12)	C7—C16—H16F	109.5
C7—C8—C9	118.62 (11)	H16A—C16—H16F	136.2
C7—C8—C15	120.00 (11)	H16B—C16—H16F	75.0
C9—C8—C15	121.38 (12)	H16C—C16—H16F	37.3
C10—C9—C8	122.48 (12)	H16D—C16—H16F	109.5
C10—C9—O1	122.14 (11)	H16E—C16—H16F	109.5
C8—C9—O1	115.37 (11)	C8—C15—H17A	109.5
C9—C10—C5	118.77 (11)	C8—C15—H17B	109.5
C9—C10—C4	120.97 (11)	H17A—C15—H17B	109.5
C5—C10—C4	120.26 (11)	C8—C15—H17C	109.5
C2—C11—H11A	109.5	H17A—C15—H17C	109.5
C2—C11—H11B	109.5	H17B—C15—H17C	109.5
H11A—C11—H11B	109.5	C8—C15—H17D	109.5
C2—C11—H11C	109.5	H17A—C15—H17D	32.1
H11A—C11—H11C	109.5	H17B—C15—H17D	133.5
H11B—C11—H11C	109.5	H17C—C15—H17D	79.9
N13—C12—C2	179.29 (15)	C8—C15—H17E	109.5
C5—C14—H14A	109.5	H17A—C15—H17E	79.9
C5—C14—H14B	109.5	H17B—C15—H17E	32.1
H14A—C14—H14B	109.5	H17C—C15—H17E	133.5
C5—C14—H14C	109.5	H17D—C15—H17E	109.5
H14A—C14—H14C	109.5	C8—C15—H17F	109.5
H14B—C14—H14C	109.5	H17A—C15—H17F	133.5
C5—C14—H14D	109.5	H17B—C15—H17F	79.9
H14A—C14—H14D	137.6	H17C—C15—H17F	32.1
H14B—C14—H14D	40.3	H17D—C15—H17F	109.5
H14C—C14—H14D	72.0	H17E—C15—H17F	109.5

### Hydrogen-bond geometry (Å, °)

**Table:**

*D*—H···*A*	*D*—H	H···*A*	*D*···*A*	*D*—H···*A*
O15—H15···N13^i^	0.84	2.23	3.013 (2)	155

Symmetry codes: (i) *x*−1/2, −*y*+1/2, −*z*.
